# Analysis of ethical considerations of COVID‑19 vaccination: lessons for future

**DOI:** 10.1186/s12910-023-00969-y

**Published:** 2023-10-27

**Authors:** Roya Malekzadeh, Ghasem Abedi, Arash Ziapour, Murat Yıldırım, Afshin Amirkhanlou

**Affiliations:** 1 Department of Public Health, Faculty of Health, Mazandaran University of Medical Sciences, Sari, Iran; 2 Cardiovascular Research Center, Health Institute, Imam-Ali Hospital, Kermanshah University of Medical Sciences, Kermanshah, Iran; 3 Department of Psychology, Faculty of Science and Letters, Agri Ibrahim Cecen University, Ağrı, Türkiye; 4 Graduate Studies and Research, Lebanese American University, Beirut, Lebanon; 5 General Practitioner, Shohada Hospital, Mazandaran University of Medical Sciences, Sari, Iran

**Keywords:** Vaccination, COVID-19, Coronavirus, Vaccine, Ethics, Public health emergency

## Abstract

**Background:**

Since the beginning of the COVID-19 pandemic, different countries sought to manufacture and supply effective vaccines to control the disease and prevent and protect public health in society. The implementation of vaccination has created many ethical dilemmas for humans, which must be recognized and resolved. Therefore, the present study was conducted to analyze the ethical considerations in vaccination against COVID-19 from the perspective of service providers.

**Methods:**

The present qualitative research was conducted in 2022 in the north of Iran. The participants included 23 health workers with at least five years of work experience and members of the COVID-19 vaccination team. The data were initially collected through systematic semi-structured interviews, then snowball sampling and finally continued until data saturation. The next steps were transcription of interviews, identification of meaning units, coding, categorization based on similarity and symmetry, extraction of themes and the analysis of themes through content analysis.

**Results:**

The analysis of participants’ experiences led to the extraction of five main categories of themes and fifteen sub-categories of the ethical considerations of COVID-19 vaccination. Safe and standard vaccine production, vaccine supply, fairness, respect for autonomy, and accountability were the main categories. The subcategories included compliance with scientific and ethical procedures, effectiveness and profitability of vaccine, absence of severe adverse effects, allocation of resources for vaccine supply, vaccine availability, diversity and comprehensiveness of alternative vaccines, vaccination prioritization, prioritization of the vulnerable populations of society, autonomy of patient (equal rights), autonomy of community, autonomy of service providers, reporting correct information, reporting vaccine side effects, public trust and acceptance.

**Conclusion:**

The health system managers should be adequately prepared to solve the ethical problems posed by COVID-19 vaccination. Therefore, it is recommended to avoid haste in vaccination and pay more attention to vaccination safety standards, provide sufficient resources for a comprehensive vaccine supply, pay close attention to collective interests versus individual interests, and meet community needs.

## Background

On December 29, 2019, doctors in hospitals in Wuhan, China noticed unusual cases of patients with pneumonia. Only then the unusual prevalence of the virus was reported in different places [1]. Finally, on January 9, 2020, the World Health Organization (WHO) announced the cause of this disease to be a new coronavirus named nCoV-2019 [2]. After the increase in infected cases and the global spread of the virus, on January 30, 2020, the WHO announced the spread of the new coronavirus as the sixth cause of public health emergency worldwide, a threat to all countries [3]. Coronavirus (COVID-19) is a large family of viruses that may cause respiratory infections from colds to more severe diseases such as MERS and Sars [4]. The symptoms of the virus vary from mild to severe [5]. The COVID-19 virus mainly causes respiratory disorders with symptoms ranging from mild disease with muscle pain, sore throat, cough, fever, anosmia and diarrhea [5–9] to moderate to severe symptoms such as acute respiratory distress, multiple organ failure and finally mortality [10].

Different approaches have been employed to deal with the spread of infectious diseases. Countries take a range of legal measures and mandatory interventions such as mandatory vaccination, mandatory medical examination, quarantine, isolation, or arresting the infected [11]. Some experts encourage compulsory treatment or vaccination against infectious diseases and generally hold the view that the need for compulsory medical treatment for infectious disease or the need for compulsory vaccination of people against infectious disease is both natural and necessary. Another population considers an unjustified violation of the patient’s right to independence and autonomy as unethical and contrary to individuals’ rights, even if there is extensive evidence of the benefits of the vaccine [12]. However, the COVID-19 crisis has turned the issue of mandatory vaccination into a controversial issue [13]. While the law plays an important role in protecting individuals against unjustified interference with individual rights, the health interests of the community can sometimes be so strong or the threat to health so great as to warrant even coercive action [14]. Therefore, mandatory measures to protect public health, such as plans to prevent or control disease, have been controversial and challenged the relationship between the government and the patient and the conflict between individual independence and public interest [15]. To manage the COVID-19 crisis in Iran, restrictive measures were taken, such as closing businesses, limiting the number of employees at work, as well as reducing the attendance hours of government employees, closing schools, social distancing in means of transportation and public places, and finally the program COVID-19 vaccination. Due to the limited number of COVID-19 vaccines in Iran, the vaccination program was based on prioritization. First, it was implemented for the treatment staff, then people with underlying diseases and elderly people over 60 years old, and then other people. The expansion of vaccination coverage was pursued through information and advertising to encourage people to get the vaccine, and then through the restriction of social services for people without vaccines. Forcing government employees to receive vaccines, preventing travel for people without vaccine cards, and not providing insurance support for people suffering from the COVID-19 disease without vaccines were among Iran’s policies in the management of Corona. Although these policies were not fully implemented [16].

Today, vaccination is essential to deal with infectious diseases [17]. Mandatory vaccination is considered a preparedness strategy during the outbreak of a severe and vaccine-preventable epidemic disease [13]. Vaccines have reduced the mortality rate of epidemics globally. The WHO estimated that 80% of infectious diseases in the world are related to diseases accounting for the death of more than 20 million people worldwide [17]. Therefore, the vaccine plays a key role in controlling infectious diseases and is a cost-effective way to control it [18]. People all over the world are living in fear of COVID-19 because they have lost their family members and loved ones due to the disease [19]. As of January 2022, more than 330 million cases of COVID-19 have been reported by the WHO, with more than 5,500,000 mortalities [9]. The real figure is probably significantly higher. It has also caused major disruptions to the global economy, with the World Bank estimating that the global recession caused by COVID-19 has been surpassed only by the two world wars and the Great Depression of the past century and a half (17). Nearly 50% of 3.3 billion global workers are at risk of losing their family life [2]. Considering the impact of COVID-19 on the social and individual level and the possibility of the emergence of more dangerous mutations, it seems logical to use mandatory vaccination against pandemic threats [15].

Even before the current pandemic, vaccine hesitancy was a familiar phenomenon, and the WHO recognized it among the top ten global health threats in 2019 [20]. Until today, several COVID-19 vaccines have been approved for use and prescribed in the countries of the world [21]. The process of vaccine production to consumption faces special ethical issues [22]. The most common reasons for concerns about vaccination are related to vaccine safety and mistrust of the pharmaceutical sector [23]. Vaccine uptake is also related to trust in the government [24]. These concerns are partially caused by false, incompetent or unethical reporting of fake scientific findings [25]. Naturally, after discovering an efficient formulation for vaccination, few countries will be able to produce it massively, and other countries in the world will need to buy it from certain others; therefore, there is competition in production, price setting and the possibility of commercialization of the matter. In developing countries with lower incomes, it is not possible to purchase and use vaccines. Within the countries, many vulnerable groups may not be able to purchase the vaccine. Fair rules and regulations make it incumbent that vulnerable and endangered populations be vaccinated [22]. The results of the study by Pourshahri (2022) showed that two factors of living with people at risk (88.5%) and respect for the rights of others (80.9%) were the most important reasons for accepting and worrying about the side effects of the vaccine (63%). Worrying about the content and lack of knowledge about the effectiveness of the vaccine (45.3%) were mentioned as the most important reasons for not accepting the COVID-19 vaccination [17]. The results of the study of Tehrani (2021) showed that Individuals may for some reason resist vaccination. For example due to, the confusion caused by mass media information, public distrust, the proposed relationship between vaccination and the development of certain diseases, and finally low death rate due to covid 19 in some groups, especially young and healthy individuals [17].

When the community is faced with public health threats, the most recent of which is the SARS-CoV-2 (COVID-19) virus, ethical challenges become controversial [26]. Corona vaccine, as the only hope to control this fatal disease, entered the healthcare domain and brought with it many ethical challenges. The challenges posed by COVID-19 are new to the modern world and can affect the global economy and society for many years to come [27]. Therefore, in addition to observing all the scientific procedures, the health system managers should also be adequately prepared to solve ethical problems and make ethical decisions. They should consider the merits and demerits of the vaccine for each individual, in contrast to those of not vaccinating the society. Therefore, the present research was carried out to investigate the ethical considerations of COVID-19 vaccination.

## Methods

### Design of the study and selection of participants

The current qualitative research used a content analysis approach. The qualitative content analysis approach was used to explore the experiences of the ethical considerations of COVID-19 vaccination. The research population consisted of healthcare workers (vaccination health experts within the community) in health centres of northern Iran (Mazandaran province) in 2022. The sampling method was first purposive and then snowball. The initial individuals who were purposively selected and met the inclusion criteria suggested the next participants. The number of interviews continued until data saturation. In this research, data saturation occurred when 23 health experts were interviewed. To collect the experts’ opinions, semi-structured individual interviews were held. Besides, the interviews were conducted based on some general questions (Table 1). The interview guide was developed specifically for this study.

The inclusion criteria were health care workers with at least 5 years of work experience in health centres of Mazandaran province, members of the public vaccination team, having the ability to express their experiences. The exclusion criterion was the presence of any disabling factor in describing caregiving experiences (e.g., inability to speak). The researchers tried to observe maximum variety in selecting the participants. A semi-structured interview was used to collect data. Having decided on the place of interview (a convenient and quiet room) based on the purpose of the study and inclusion criteria, the researchers selected the participants. Having obtained informed consent and ensured the participants of the confidentiality of their information, the researchers went on with the face-to-face interview and asked questions. The interviews were audio-recorded upon the interviewees’ consent. During the interviews, open questions were asked and the participants were allowed to describe their experiences. They were asked, “What comes to your mind when you hear the name of coronavirus vaccination?“ “Tell us about your experiences with the COVID-19 vaccination?“, “Tell me the ethical considerations observed concerning COVID-19 vaccination?“ During the interview, the focus was on the cases to drive the patient towards his/her experience of the ethical considerations of COVID-19 vaccination. When there was a need to further clarify certain information, more detailed questions were used. Finally, the participants were asked to provide further explanation in describing the ethical considerations of COVID-19 vaccination. The rest of the follow-up and exploratory questions were asked based on the data provided by the participant, to clarify the concept and deepen the interview process. The sampling continued until data saturation or until no new data were extracted as the interview continued. The researcher tried to be an active listener as far as possible. The interview took between 30 and 40 min and varied depending on the participant’s condition. During the interview, the focus was on the cases to drive the patient towards his/her experience of the ethical considerations of COVID-19 vaccination.

For data analysis, summarizing the information was done immediately after each interview. The summaries included the interviewer’s perception of the main ideas raised in the interview. Then, the audio recordings and the transcripts were revisited for content. In the end, the manuscripts were checked with the content of the tape. Granheim and Lundman’s qualitative approach was used for content analysis [28], which was as follows:

1- The researcher made the interviews in written form and studied them several times to develop an in-depth understanding. 2- All interviews were considered as a unit of analysis. 3- Paragraphs, sentences or words were considered as meaning units. A meaning unit is a set of words and sentences related to each other in terms of content and summarized and placed side by side according to the similarity of content. 4- Semantic units reached the level of abstraction and conceptualization according to the latent concept in them and were then coded. 5- The codes were compared with each other in terms of similarities and differences and were categorized into further abstract groups with specific labels. 6- Finally, by comparing the categories with each other, and detailed and deep reflection, the latent content in the data was introduced as the running theme of the study.

**Table 1:** Interview questions

1. Tell me about your experiences with the covid-19 vaccination? 2. Tell me what ethical issues and considerations did you encounter during the covid-19 vaccination? 3. How was the implementation of the Covid-19 vaccination program in the country from your point of view and according to your experiences? 4. What were people most worried about at the time of vaccination? 5. What questions did people ask most during the time of the vaccine? 6. What were the opinions of the people who applied for the vaccine injection about the Covid-19 vaccine? 7. What was your experience regarding “compulsory covid-19 vaccination and imposing restrictions on people without vaccines”? 8. Considering the restrictions on the number of vaccines in the two years since the start of Corona in the country; Tell me your experiences about choosing people for the vaccine?	

For the accuracy and robustness of the study, Guba and Lincoln’s criteria were used [29]. The researcher tried to increase the credibility of the research by long-term sufficient participation and interaction with the participants, collecting valid information and verifying the participants’ information. Also, to increase the reliability of data, step-by-step replication and data collection and analysis, the review by the supervisor, consultant and experts was performed. To increase the data verifiability criteria, the approval of university professors and their additional comments were used. To increase transferability, the participants’ quotes were stated in the same way as originally mentioned. It was attempted to provide rich descriptions of the research report to evaluate and increase the applicability of research in other fields so that other researchers can understand the ethical considerations of COVID-19 vaccination and apply them.

To comply with ethical considerations and protect the participants’ rights, the researcher, got a permission letter from the Deputy of Research and Technology of Mazandaran University of Medical Sciences. Then, he introduced himself to the participants and explained the objectives of the study. The participants were asked for informed consent. They were assured that the interviewed materials were completely confidential and anonymous as emphasized in the ethics guide in the publication of works of research.

### Ethics

This study was approved by the Ethics Committee at Mazandaran University of Medical Sciences (IR.MAZUMS.REC.1401.11796) and was guided by the Declaration of Helsinki’s ethical principle [30]. In addition, permissions from respective institutional gatekeepers were obtained to access potential participants from different institutions as required. All participants signed an informed consent form stating that they understood the nature and purpose of the research and that they agreed to their interview being recorded. All personal and institutional identifying data were removed from the interview transcripts before coding and analysis.

## Results

In the qualitative study, the opinions of 23 healthcare providers (providers of public vaccination services) in the health centres of Mazandaran province were included. They consisted of 7 doctors, 8 nurses, 4 hospital heads and managers, and 4 public health experts implementing the COVID-19 vaccination program. 59% of participants were female and 41% were male. Nearly half of the participants (43.5%) had a doctorate and the rest (56.5%) had a bachelor’s and master’s degree (Table 2). From the rich and in-depth description of the participants, 320 primary codes were extracted. Using performance evaluation framework analysis, five main categories fifteen sub-categories and 45 items were extracted (Table 3).

**Table 2:** Participants’ demographic information to explain the ethical considerations of COVID-19 vaccination

Variable	Level	F(%)
Sex	Female	13(56.5)
	Male	9(43.5)
Education	Bachelor’s degree	8(35)
	Master’s degree	4(17)
	Ph.D.	11(48)
Occupation	Doctor	7(30.4)
	Nurse	8(34.8)
	Manager	4(17.4)
	Health expert	4(17.4)
Age	20–30	2(8.6)
	30–40	4(17.4)
	40–50	13(56.6)
	> 50	4(17.4)
Work experience	< 10	3(13)
	10–15	4(17)
	15–20	11(48)
	20–25	3(13)
	> 25	2(9)

**Table 3:** Extracted categories of ethical considerations of COVID-19 vaccination from the service providers’ perspective

Category	Sub-category
Safe and standard vaccine production	Compliance with scientific and ethical procedures Effectiveness and profitability of the vaccine Absence of severe adverse effects on health
Vaccine supply	Allocation of sources for vaccine supply Vaccine availability Diversity and comprehensiveness of alternative vaccines
Fairness	Vaccine fair distribution Vaccine fair prioritization Prioritization of vulnerable populations of society
The significance of autonomy	Autonomy of patient (equal respect) Autonomy of community Autonomy of health service providers
Accountability	Reporting correct information Reporting vaccine side effects Public trust and acceptance

### Safe and standard vaccine production

According to the majority of research participants, the production of standard and safe vaccines is necessary. It Is incumbent on the health system and the government. Metaphors such as obtaining emergency production permits, novelty of vaccine type, and unclear long-term side effects show public concerns. The production of safe and standard vaccines was a main theme with 3 categories: compliance with scientific and ethical procedures, effectiveness and profitability of the vaccine, and absence of severe adverse effects.

#### Compliance with scientific and ethical procedures

Almost all participants mentioned compliance with scientific and ethical procedures among their experienced considerations. The participants pinpointed gaining permission for the emergency production of the COVID-19 vaccine without going through the scientific procedures and conducting the correct clinical trial as an ethical concern of COVID-19 vaccination. From the participants’ perspective, “Many people were waiting for the advent of safe and standard vaccines that passed the correct pre-clinical and clinical stages. As they mentioned, the existing vaccines had not gone through the official, scientific procedure before gaining the approval of the WHO” (P14). Another participant said, “Fear of the virus should not cause haste and neglect vaccine safety standards” (P19). Another mentioned, “In addition to prioritizing vaccine production for public use, health authorities should pay attention to all scientific considerations in vaccine production and be prepared to solve ethical problems” (P6).

#### Effectiveness and profitability of the vaccine

Most participants listed the effectiveness of vaccine shots among the ethical considerations of COVID-19 vaccination. One participant stated: “Several people who received the AstraZeneca vaccine had serious complications such as Coronavirus infection or were hospitalized” (P18). Or another participant maintained, “I even know people who had been vaccinated but after a CT scan and showed symptoms of lung involvement after the shot” (P14). Another participant stated that “there are doubts about the effectiveness and usefulness of the vaccine. The possibility of life-threatening and harmful health effects causes fear and concern for the use of domestically produced vaccines and threatens the reception” (P19 and P4).

#### Absence of severe adverse effects

Most participants stated that when the national vaccination program began, most people perceived the program with fear and showed concerns about complications such as sudden death, getting infected by the vaccine, feeling severe pain or paralysis of limbs, etc. A participant said, “In our hospital with 80 specialists and 10 general practitioners, none were willing to be vaccinated. The head of the hospital mediated for vaccination and invited 10 veteran doctors. He aimed to encourage others to warmly receive the vaccination” (P7). Another participant said, “Concerns about complications were so great that the representatives of doctors and nurses asked the head of the hospital to be the first person to get the vaccine. If nothing happened, the rest of the staff would also go for it” (P1 and P20). “Online space was also influenced. The video clips published on Telegram and WhatsApp showed strange side effects caused by the COVID-19 vaccination of in infect some people” (P2). Another participant said, “Because the health workers were chosen as the first beneficiaries of vaccination, they felt they were chosen for laboratory work to investigate the effects and side effects of the vaccine” (P18).

### Vaccine supply

The participants believed that after finding the formulation of the COVID-19 vaccine because few countries can produce it massively, there will be serious competition for supplying vaccine in different communities. Countries such as Iran, which is facing sanctions, will be able to supply the vaccine certain challenges. Vaccine supply was a category obtained with 3 sub-categories of resource allocation for vaccine supply, vaccine availability, vaccine diversity and comprehensiveness.

#### Allocation of resources for vaccine supply

The participants believed that the government should feel an obligation to maintain and improve public health in society; therefore, it is necessary to take full advantage of all the country’s resources to provide timely high-quality vaccines. One participant stated, “The governance of the health system can be analyzed based on their reaction to key issues threatening people’s health in society” (P8). Another participant stated, “Timely supply of vaccine could reduce the spread of the pandemic in the country and reduce the mortality rate” (P21 and P11). Or, another participant mentioned, “Using all resources, including political and diplomatic resources, financial resources, and allocating appropriate budgets to sign contracts with vaccine manufacturing companies can to a large extent reduce the severity of the pandemic and bring peace to society” (P21).

#### Vaccine availability

The participants stated that “at the beginning of the vaccination program, a limited number of vaccines were available to the centres for injection. Each time, a quota was allocated to the country’s vaccination centres, which was not enough for a large number of applicants, although in the second year of the vaccination program, the majority of people received their shots, and this deficiency in availability was less noticeable” (P4, P5, P10). One participant said, “Sinopharm vaccine has been provided to 200 people, while those who received Sputnik in the first round of the vaccine were waiting for the expert opinions in the ministry about the compatibility between two different vaccines to ensure public safety. The specific quota for health centres was inadequate (P2 and P13).

#### Diversity and comprehensiveness of the vaccine

Most participants pinpointed the limited diversity of the COVID-19 vaccines in the country and the lack of vaccine supply according to people’s expectations and the compulsion to use the vaccine among ethical considerations of national vaccination. For example, two participants stated, “In the first year of vaccination, there were no different vaccines to choose from, and due to the limited supply and production of vaccines, only a certain type of vaccine was imported to the country each time, and there was no other choice people could make” (P2 and P8). Another participant stated, “Despite the restriction of the vaccine in the country, even the same quota was not warmly received. People were looking for their desired vaccines” (P2). Still, another participant stated, “The type of vaccine that was imported was not the type people were expecting, so a significant percentage of vaccines that were imported were not being used” (P13 and P11).

### Fairness

#### Vaccine fair distribution

As the participants perceived, “Whenever people feel the whole community is considered equal in the eyes of the health system administrators and there is no discrimination, a sense of peace and justice arises” (p 14). Another participant stated, “The fear of the coronavirus and its deadly complications that were around in society in the first year caused many people to question the lack of vaccine and the delayed supply” (P9)., another participant mentioned, “All provinces of the country did not receive the same quota according to their population, and they doubted the fairness of the vaccine coverage of various types they had heard about” (P17).

#### Vaccination prioritization

Many participants were satisfied with the prioritization of people in society to receive vaccines according to age and occupation. One participant said, “In the first year of vaccination, quotas were reserved for health service providers and gradually for the general public by prioritizing and classifying target groups such as the elderly (according to their birth year, age, comorbidities and incurability of disease)” (P22). As some participants experienced, “in a pandemic condition of a disease like COVID-19 and the concomitant lack of a vaccine or any medicine to control or treat the disease, special planning is needed to allocate this limited amount of medicine or vaccine to the target population according to scientific and ethical criteria and standards” (P3). “Whenever the public feels there is no discrimination among people in accessing the vaccine, and prioritization is only based on scientific expert opinion, everyone can be provided with the vaccine at specific time intervals” (p 14).

#### Prioritization of the vulnerable populations of society

Most participants deemed it necessary to pay attention to vulnerable populations of society in protection and immunity from COVID-19. The participants stated, “As different studies and information from the media showed, some people were more prone to the deadly effects of the coronavirus and had a comorbidity, so they had to be prioritized by the government” (P8 and P3)., another participant said, “Patients who should be immediately supported by the vaccination program due to a comorbidity or incurable disease were covered by earlier reception of the vaccine” (p 22). “In the first year of the pandemic, a large number of people with special diseases were infected or hospitalized as the rate of coverage for the vulnerable population was significantly lower than expected” (P19).

### The significance of autonomy

The analysis of data obtained from individual and collective interviews showed the significance of autonomy to participants. This category included three sub-categories, the autonomy of patient, autonomy of community and the autonomy of health providers.

#### Autonomy of patient (equal respect)

As the participants stated, “It is not fair for people to say their lives are theirs and they decide not to get vaccinated and immune” (P1). “Because disease control is achieved by immunizing all people, it is not appropriate for people to refrain from vaccination and perceive it as a matter of personal affair to decide on” (P7). “The right to choose and individual freedom are respected to the extent that they do not threaten others when only the individual him/herself faces the consequences of his/her decision” (P5). Also, another participant said, “I agree with the mandatory vaccination, but people should be given the right to choose the type of vaccine, not just be forced to take a specific one” (p 17).

#### Autonomy of community

One participant stated that “during the epidemic of a contagious disease, the whole society is affected by its adverse effects, so the entire society should be respected” (P1). He emphasized the importance and priority of public health over the individual. Other participants also addressed this issue, for instance, “COVID-19 pandemic caused a lot of economic and social losses to different groups of society. It damaged the national education system, and maximum coverage of vaccination can prevent these losses (P13). Another participant stated that “vaccination is considered a social duty and if people are free to be vaccinated, the health of the society will be endangered” (P12).

#### Autonomy of healthcare providers

As most participants agreed, the government should include incentive and punitive laws for maximum coverage of vaccination nationwide. Considering that the vaccine will be effective in controlling the coronavirus when a significant percentage of people are vaccinated, the government should set certain limits on social services for those who deliberately avoid vaccination” (P14). Also, another participant admitted, “The government can enact a law to make people who avoid vaccination and transmit the disease pay high costs of treatment themselves with no support by the insurance (p 22).

### Accountability

Another finding of the data analysis was accountability. This category included the three subcategories of reporting correct information, reporting vaccine side effects and public trust and acceptance.

#### Reporting correct information

One participant commented, “The cyberspace was very misleading. There were images and video clips claiming that the officials took foreign shots while recommending the locally manufactured to people” (P5). The participants also stated that “when the attention of the country’s health system is devoted to immunity through vaccines, complete, honest and reliable information should be provided to people” (P15). “In addition to the fact that the benefits of the vaccine are constantly emphasized by the media, the potential side effects or ways to deal with the side effects should be revealed by doctors and experts so that people can trust them” (P18). By providing correct and timely information to the society, doctors can help them make the right decision” (P3). To deal with this misleading role of cyberspace, the Ministry of Health should be more active“(P16). However, unfortunately, cyberspace misled people and the government was not as responsive as it should (P14).

#### Reporting vaccine side effects

The participants stated that worrying about and sometimes observing the side effects of the vaccine in cyberspace or official media was a major concern of society. Many participants believed “the side effects of vaccines are not revealed to people not to discourage them from vaccination, while they were aware it was not ethical or professional” (P1). The side effects of the vaccine were not well communicated so that people would not worry” (P16). “People who visited health centres or were informed about the side effects of the vaccine did not receive any persuasive response in the short term, and a large number of people believed the information they provided was soon to be exploited by those in charge of research purposes or personal benefits” (P23).

#### Public trust and acceptance

The participants believed that the unprecedented nature of the disease and its epidemic, on the one hand, and the unprecedented focus of the world news on this issue, on the other, created too much concern and fear in society. Contradictory news about the pandemic or the dual policies of the government regarding vaccination made people lose their trust. On the one hand, the side effects of foreign vaccines were highlighted in media, but there was no mention of the side effects of domestic vaccines” (P11). “On the other hand, they locked down everywhere to break the chain of infection, yet travel was free and the country’s roads were open“(P1). “Not telling the truth about the adverse effects of the vaccine, not getting the approval of international assemblies for nationally manufactured vaccines were serious ethical concerns” (P16). The participants stated that “to fulfill the vaccination program, the government should honestly elaborate on the stages of vaccine production, valid approvals, etc. It should consider people’s concerns and gain their trust to communicate properly with society, and not by mere coercion” (p 21).

## Discussion

The present study led to the identification of the ethical considerations of COVID-19 vaccination in five main categories: “production of safe and standard vaccine”, “vaccine supply”, “fairness”, “significance of autonomy” and “accountability”. According to the results, compliance with scientific and ethical procedures, efficacy and profitability of the vaccine and the absence of severe adverse effects on health are among the ethical considerations of safe and standard vaccine production. In this study, the service providers maintained although the production of a standard and safe vaccine is necessary, metaphors such as gaining emergency production permits, the novelty of the vaccine type, and the unclear long-term side effects show the concern of the society with this regard. Safe and standard vaccines that have passed the correct scientific steps have an important preventive role in public health [31, 32]. Naturally, the significance depends on the effectiveness of vaccination in terms of fewer side effects and the absence of severe life-threatening side effects [33–35]. It is noteworthy that vaccines are manufactured to prevent infectious diseases and to increase the immunity level of society. Therefore, people in society should not be prone to the severe side effects of vaccination [36, 37]. Health service providers should be reminded that the advantages of public health measures and interventions are more than the disadvantages for the public [38]. The results of Pourshahri’s study showed that factors such as the fear of side effects of the vaccine, concern about the content of the vaccine and lack of knowledge about its effectiveness are related to the non-acceptance of the COVID-19 vaccination [39]. Gaduth’s study showed that 35% of health workers are worried about the side effects of the COVID-19 vaccine [40]. In Qatan’s study, 26.73% of people refused to get the vaccine due to the fear of the adverse effects [41].

As the present findings showed, allocation of resources for vaccine supply, vaccine availability, vaccine diversity and comprehensiveness are among the ethical considerations of vaccine supply. In this study, service providers stated that allocating resources to manufacture the vaccine that society needs is a duty of the government. Metaphors such as the existence of authentic vaccines, people’s right to choose the vaccine type, and not being forced to get a specific type due to the limited availability of others show the need to adequately attend to the diversity and comprehensiveness of alternative vaccines in society. Naturally, after discovering an efficient formulation for public vaccination, few countries will be able to produce it massively, and other countries will need to purchase it from the manufacturing countries; therefore, it is essential to find a way to supply the vaccine for the public [22], which is considered a sign of the good governance of the health system.

As the results showed, a fair distribution of vaccines, a fair prioritization process and prioritization of vulnerable populations of society are among the ethical considerations of fairness. In a fair allocation of vaccines, the moral principles of fairness and utility become operative. The principle of fairness guides health policymakers to provide vaccines fairly for all members of society, and the principle of utility directs them to use resources in a way that the society takes maximum advantage of the program [42]. In some countries, due to the proposed price of the vaccine, many vulnerable populations may not be able to pay for the vaccine. If the cost of vaccination is borne by the people themselves, not affording to pay for vaccination may be a problem for some. It can prevent low-income vulnerable populations from getting the vaccine [22]. therefore, the governments have to support the vulnerable populations of society. By vaccinating them, both the death rate will decrease and society will reach a basic immunity rate.

As the present study showed, the autonomy of the patient (equal respect), autonomy of the community, and health service provider are among the ethical considerations of the significance of autonomy. Today, autonomy of patients, which means respecting the patient’s independence and individual freedom, is a major principle accepted in modern medicine. Many of the principles of medical ethics, such as informed consent, respect for privacy, confidentiality and integrity, are based on the same principle [43, 44]. Concerning any type of healthcare service in the health system, different parties are involved in decision-making, each with its right to autonomy (patient, society, service provider) [45]. According to the principle of individual autonomy, each member of society has the right to be informed of sufficient information about the vaccine and to decide whether or not to get the vaccine according to their health conditions. During the epidemic of infectious diseases that affect the entire society due to widespread adverse effects, it is necessary to somehow replace the principle of autonomy with the principle of equal respect. According to the medical ethics philosophy, autonomy is used to the extent that no harm is done to others. Quarantine the people and trace the contacts of the patients it is reducing the priority of the principle of respecting autonomy and operationalizing the principle of benefiting the society (society autonomy). By increasing the immunity rate in the society, giving the vaccine reduces the risk of infection in the pandemic and the death rate in the society. The epidemic of COVID-19 has caused serious damage to the health of the people of the society as well as the socio-economic functions of the people of the society, and vaccination against this disease can prevent these damages. Of course, to ensure the effect of the vaccine, a significant number of people in the community must receive the vaccine; Therefore, governments and policymakers may declare it necessary. Therefore, it is possible to consider vaccinating as a moral duty with the assumption that if all people refrain from injecting the vaccine, the safety of society will be endangered (Deontology). The goal of protecting society can justify not respecting people’s autonomy. It seems that the more serious the damage to the society, the more defensible these arguments become (Utilitarianism) [39]. According to ethical principles, the health system is in charge of making preventive interventions to promote public health and protect society from infectious diseases that may pose serious threats to the health of society. Those involved in the health system should look for a correct and suitable solution so that the individual health of society is not threatened as well as the public health of society. The results of Pourshahri’s research showed that 9% of the vaccinated population mentioned respecting others’ rights by getting vaccinated as one reason for accepting the COVID-19 vaccination [39]. In Bell’s study, vaccination for the sake of protecting oneself and others was suggested as the most common reason for accepting the COVID-19 vaccine [46]. This figure in other studies on people’s attitudes towards the COVID-19 vaccination was close to 54.7 and 65.5% [47, 48].

In light of the present findings, it can be concluded that providing correct information to people, reporting the side effects, and gaining public trust are among the ethical considerations of government accountability. Restoring trust and correct communication with society can be done in a variety of ways to face public concerns and remove misconceptions. Adequately informing society of the consequences of avoiding vaccination, the possibility of infecting vulnerable populations and increasing the mortality rate will be truly helpful [49, 50]. Low awareness is a main barrier to public vaccination coverage [51]. Reporting the side effects of vaccines is also sometimes the main concern of society [52] because sometimes society receives false reports of the side effects of a vaccine. The health system, including health care providers, is supposed to provide reliable medical advice and proper public health care [54, 55]. The availability of research-based information that has not yet been proven, is constantly changing or has not been properly and scientifically managed, increased mistrust during the pandemic. Some research on health workers showed the lack of transparency in information available to the public about a vaccine is considered the main reason for avoiding vaccination [40]. The existing literature shows that public concern is due to the lack of knowledge about the content and efficacy of the vaccine, which itself can be a reason for the public lack of trust in vaccination [39]. In other words, to convince communities to accept vaccination, hard evidence is needed about the safety and effectiveness of the vaccine. This claim is very close to the views of the participants in the present study.

## Conclusion

In this research, the production of safe and standard vaccine, vaccine supply, respect for autonomy and reaction to people’s concerns were raised as the main ethical considerations of COVID-19 vaccination. Raising public awareness and knowledge of the effectiveness of vaccines nationally and efforts to reduce the fear of side effects require planning and investment at the macro level of the country. Also, attempts should be made to strengthen people’s desire to protect others by vaccinating themselves and increasing people’s correct information through networks and social media.

## Data Availability

The datasets generated and/or analysed during the current study are not publicly available due to consent not being obtained from participants for this purpose but are available from the corresponding author on reasonable request.

## Abbreviations

COP: COVID-19 Pandemic
EC: Ethical considerations
QR: Qualitative research
